# Development, genetic and cytogenetic analyses of genetic sexing strains of the Mexican fruit fly, *Anastrepha ludens *Loew (Diptera: Tephritidae)

**DOI:** 10.1186/1471-2156-15-S2-S1

**Published:** 2014-12-01

**Authors:** Cristina Silvia Zepeda-Cisneros, José Salvador Meza Hernández, Víctor García-Martínez, Jorge Ibañez-Palacios, Antigone Zacharopoulou, Gerald Franz

**Affiliations:** 1 Moscafrut Program. Agreement SAGARPA-IICA. Programa Nacional Moscas de la Fruta. Metapa de Domínguez Chiapas, México C.P. 30860; 2 University of Patras, Patras, Greece; 3 Insect Pest Control Laboratory, Joint FAO/IAEA Programme of Nuclear Techniques in Food and Agriculture

**Keywords:** *Anastrepha ludens*, Genetic sexing strains, Polytene chromosomes, Mitotic chromosomes, Sterile insect technique

## Abstract

**Background:**

*Anastrepha ludens *is among the pests that have a major impact on México's economy because it attacks fruits as citrus and mangoes. The Mexican Federal government uses integrated pest management to control *A. ludens *through the Programa Nacional Moscas de la Fruta [National Fruit Fly Program, SAGARPA-SENASICA]. One of the main components of this program is the sterile insect technique (SIT), which is used to control field populations of the pest by releasing sterile flies.

**Results:**

To increase the efficiency of this technique, we have developed a genetic sexing strain (GSS) in which the sexing mechanism is based on a pupal colour dimorphism (brown-black) and is the result of a reciprocal translocation between the Y chromosome and the autosome bearing the *black pupae *(*bp*) locus. Ten strains producing wild-type (brown pupae) males and mutant (black pupae) females were isolated. Subsequent evaluations for several generations were performed in most of these strains. The translocation strain named *Tapachula-7 *showed minimal effect on survival and the best genetic stability of all ten strains. Genetic and cytogenetic analyses were performed using mitotic and polytene chromosomes and we succeeded to characterize the chromosomal structure of this reciprocal translocation and map the autosome breakpoint, despite the fact that the Y chromosome is not visible in polytene nuclei following standard staining.

**Conclusions:**

We show that mitotic and polytene chromosomes can be used in cytogenetic analyses towards the development of genetic control methods in this pest species. The present work is the first report of the construction of GSS of *Anastrepha ludens*, with potential use in a future Moscafrut operational program.

## Background

*Anastrepha ludens *has a major effect on México's economy because it attacks fruits as citrus and mangoes. In 2008 the exports of these two fruits only to the U.S. had a value of 121.8 million US dollars [1]. The Mexican federal government uses integrated pest management to control *A. ludens *through the Programa Nacional Moscas de la Fruta (SAGARPA-SENASICA). The sterile insect technique (SIT) is one of the main components of this program and involves mass-rearing and release of millions of sterile insects to reduce the birth rate in the target population [2-4]. This method has been successfully applied to control pests such as the screwworm fly, *Cochliomyia hominivorax *(Coquerel) and the Mediterranean fruit fly *Ceratitis capitata *(Wiedemann) [5,6]. The main benefits of this method are that it is species-specific, does not generate biological or chemical pollution and is therefore environmentally friendly. The efficiency of the sterile insect technique increases if only males are released [7].

GSS have been constructed, in particular for *Ceratitis capitata*, and are used successfully in operational programs, demonstrating the following major advantages: 1. Economic savings in mass-rearing, irradiation, packing and release, 2. Increased efficiency in the field because sterile males compete better for wild females in the absence of sterile females 3. Increased fruit quality, avoiding damage caused by oviposition attempts by sterile females, and 4. In combination with a GSS, female-specific attractants in traps can be used thereby avoiding the re-capture of sterile males, reducing misidentification and simplifying and improving the accuracy of monitoring activities [8-10].

The construction of a GSS is based on two components: 1) A mutation that can be used as a selectable marker for sex separation; and 2) Y- autosome translocation linking the inheritance of this mutation to the sex, since the Y chromosome is responsible for the male sex [10,11]. In a GSS, the wild-type allele of the selectable marker is physically linked to the Y chromosome through a Y-autosome translocation, while the females carry the mutant allele in homozygous condition. It should be pointed out that Y chromosome is carrying the Maleness factor, as it was shown in *C. capitata *and this probably holds for the whole Tephritidae family [12]. The main problems affecting the usefulness of GSSs based on Y-autosome translocations are:

1) Genetic instability is due to pre-meiotic recombination in the parental male [13]. Although the recombination frequency is very low, accumulation and selection in favour of recombinants in the harsh environment of mass-rearing can lead eventually to the breakdown of the sexing system. This problem can be minimized by selecting a Y-autosome translocation where the autosomal breakpoint is close to the chromosomal location of the selectable marker [10,11].

2) Reduced product quality due to the survival of genetically unbalanced individuals resulting from adjacent-1 segregation during meiosis in the parental males [10]. This problem can be minimized by selecting a Y-autosome translocation where the adjacent-individuals die during early stages of development.

Genetic and cytogenetic analyses are necessary towards the selection of the most suitable GSS. The analysis of mitotic and polytene chromosomes provides detailed information on the structure of the chromosomal rearrangement, allowing the identification of the autosome(s) involved in the translocation and the mapping of chromosomes breakpoints. The evaluation of any new GSS is advisable before it should be used in mass-rearing [14-19].

In México, the sterile insect technique against the Mexican fruit fly *Anastrepha ludens *has been applied by releasing females and males because so far no GSS was available for that species. The development of the first genetic sexing system for this pest, based on the Y-autosome translocations and using the genetic marker *black pupae *(*bp*), is presented in this study.

Several GSS were constructed and tested, and one was selected because of its properties and potential to be used successfully in the Moscafrut mass-rearing facility.

## Results

### Genetic analysis of the *black pupae *mutation

The two reciprocal crosses, ♂*bp*^+ ^× ♀*bp *and ♂*bp *× ♀*bp^+^*, produced wild-type offspring.

**Figure 1:**
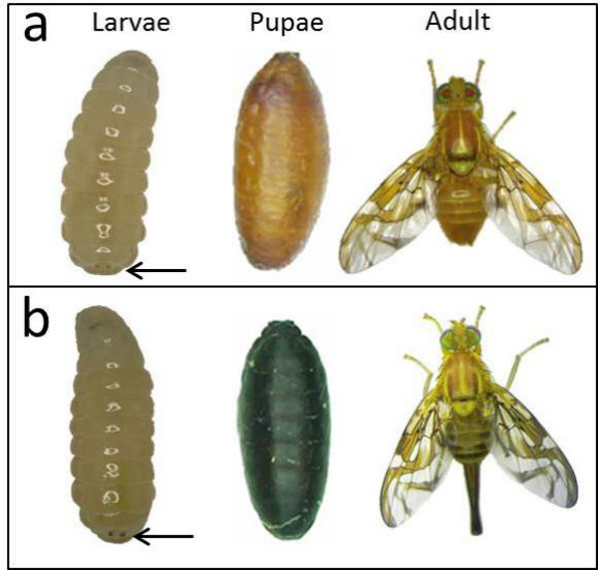
**Larvae, pupae and adults from the **GSS ***Tapachula -7 *based on the *bp *mutation**. The males can be identified in the larval stage based on the brown coloration of their anal lobes compared to the black coloration of the females. a) *wild-type *males. b) *black pupae *females. Arrows point to the anal lobes.

Inbreeding the F_1 _produced wild-type and mutant phenotypes at a ratio of 3:1 (Table 1). From these results, we conclude that the *bp *mutation is recessive and autosomal. The correct classification of the colour of the pupae was confirmed based on the pigmentation of the adults. The abdomen, thorax and wings of *black pupae *adults are darker than the wild-type flies. In addition, the *black pupae *phenotype can be identified in the larval stage based on the black coloration of their anal lobes (Figure 1).

**Table 1:** F_2 _progeny of the cross between the wild-type and the *black pupae *strain

Parents		Cage	Wild-type	*black pupae*	X^2^
				
Male	Female		(no.)	(no.)	(3:1)
Wild-type	*black pupae*	1	2,804	952	0.24
		2	2,343	738	1.80
		3	1,866	610	0.17

*black* *pupae*	Wild-type	1	2,190	668	4.03
		2	2,266	759	0.13
		3	1,761	572	0.29

X^2 ^tables = 3.84, *P *= 0.05, df = 1

### Development of genetic sexing strains

Ten genetic sexing strains were detected after screening 2,600 single male families, corresponding to a success rate of 0.38%. These strains were maintained in the laboratory for several generations. Females consistently emerged from black pupae, while males emerged from brown pupae (Figure 1).

Each strain had a specific average value of egg hatching. The strains T(Y:*bp*^+^)-7 T(Y:*bp*^+^)-3, T(Y:*bp*^+^)-1, T(Y:*bp*^+^)-10, T(Y:*bp*^+^)-6, T(Y:*bp*^+^)-2, T(Y:*bp*^+^)-8 and T(Y:*bp*^+^)-5 had the highest values, and their hatching ranged from 67.91% to 34.41%. The strains with the lowest values, T(Y:*bp*^+^)-4 and T(Y:*bp*^+^)-9 showed a hatch rate of 14.4% and 28.81%, respectively (Table 2).

**Table 2:** Evaluation during multiple generations of ten genetic sexing strains of *Anastrepha ludens*

GSS	Number of generations evaluated	Pupae (no.)		Adults (no.)				Adults (%)	Egg hatch (%)	Recombinants (%)
							
				*bp^+^*		*bp*				
					
		*bp^+^*	*bp*	♂	♀	♂	♀			
1	16	17,343	15,060	15,580	82	34	12,652	28,348	55.1	0.41
2	15	9,031	6,712	7,914	119	125	5,841	13,999	42.7	1.74
3	8	6,545	6,438	5,283	188	96	4,633	10,200	58.07	2.78
4	9	2,074	1,973	1,889	1	0	1,613	3,503	14.4	0.03
5	6	4,641	4,172	3,817	0	0	3,127	6,944	34.41	0
6	6	7,362	4,742	5,893	0	0	3,882	9,775	44.29	0
7	7	10,765	9,843	9,592	0	0	8,004	17,597	67.91	0
8	5	2,738	2,614	2,358	0	32	2,040	4,430	39.29	0.72
9	5	1,367	2,059	1,171	1	4	1,406	2,582	28.81	0.19
10	6	4,825	4,634	4,510	3	17	3,723	8,253	53.84	0.24

The ten strains analyzed also showed differences in the percentage of recombinant progeny. T(Y:*bp*^+^)-3, T(Y:*bp*^+^)-2 and T(Y:*bp*^+^)-8 had the highest values, with 2.78%, 1.74% and 0.72%, respectively. In contrast, the strains T(Y:*bp*^+^)-5, T(Y:*bp*^+^)-6 and T(Y:*bp*^+^)-7 did not show any recombinants at this level of rearing.

During this first evaluation, the strain T(Y:*bp*^+^)-7 showed a higher egg hatch as compared to the other GSS (Table 2).

### Survival test and genetic stability

The comparative analysis of the survival showed significant differences between the wild-type strain and the GSS T(Y:*bp*^+^)-7. The primary differences were observed at egg hatching (F = 135.46_2, 27_; *P*<0.0001) and in larval survival (F = 32.37_2, 27_; *P*<0.0001) while the differences in adult emergence were not significant (F = 1.69_2, 27_; *P *= 0.202) (Table 3). A comparison of the egg to adult survival between the wild-type and the translocation strain shows that T(Y:*bp*^+^)-7 is roughly 50% sterile (68.5% as compared to 39.3%, Table 3). Based on the results obtained with the medfly [20] this shows that T(Y:*bp*^+^)-7 carries a simple, reciprocal Y-autosome translocation, i.e. only one autosome is linked to the Y chromosome.

**Table 3:** Survival of the wild-type and the *black pupae *strain compared to the genetic sexing strain T(Y:*bp*^+^)-7 (mean ± SD)

Strain	Egg hatched (%)	Larval survival 3^rd ^instar (%)	Larval to pupal survival (%)	Adult emergence (%)	Egg to adult survival (%)
Wild type	94.60 ± 3.20a	73.40 ± 11.73a	98.48 ± 1.12a	94.72 ± 4.30a	68.50 ± 12.00a
*bp/bp*	91.80 ± 4.28a	76.20 ± 7.34a	93.94 ± 5.74b	91.23 ± 4.44a	65.30 ± 8.12a
T(Y:*bp*^+^)-7	63.70 ± 6.00b	43.90 ± 5.91b	98.66 ± 2.92a	91.02 ± 6.16a	39.30 ± 5.25b

The same letter after the mean values indicates that the differences between these lines are not significant.

In the first experiment to determine the genetic stability of T(Y:*bp*^+^)-7, the strain was reared with 600 to 2500 parental pairs per generation and no recombinants were observed (Table 4). However, in the second experiment where the population was increased averages of 0.025% recombinant insects were found per generation (Table 5). A third, larger scale experiment (15,000 parental pupae per generation) was set up but this time recombinants were not removed. The highest level of recombinants reached 1.5% in the eighth generation (Table 6).

**Table 4:** Progeny of the strain T(Y:*bp*^+^)-7 during multiple generations (F) with different numbers of breeders

F	No. of pairs	No. of pupae		Total pupae	Adults *bp*				Adults *bp^+^*				Total insects
						
		*bp*	*bp^+^*		♀	♂	♀ partly emerged	♀ deformed	♂	♀	♂ partly emerged	♂ deformed	
14	2,500	2,481	2,913	5,394	1,625	0	_	0	2,219	0	_	_	3,844
15	1,500	3,000	3,000	6,000	2,476	0	74	21	2,750	0	19	13	5,226
16	1,500	2,600	2,800	5,400	2,059	0	85	19	2,492	0	25	10	4,551
17	600	690	700	1,390	610	0	_	_	605	0	_	_	1,215
18	1,500	2,310	2,665	4,975	1,894	0	102	37	2,392	0	59	28	4,286
19	1,500	2,607	2,659	5,266	2,222	0	76	24	2,415	0	15	13	4,637
20	1,500	3,000	3,000	6,000	2,507	0	106	34	2,809	0	29	12	5,316

**Table 5:** Progeny of the strain T(Y:*bp*^+^)-7 during eight generations of rearing with 3000 females and 2500 males per cage (recombinants were removed each generation)

Adults *bp*				Adults *bp^+^*				Total insects	Recombinants (%)
			
♀	♂	♀ partly emerged	♀ deformed	♂	♀	♂ partly emerged	♂ deformed		
3549	0	123	19	2698	0	50	15	6281	0.00
3455	0	130	6	2802	1	39	7	6271	0.02
3916	0	64	14	2698	0	14	7	6635	0.00
3842	1	68	15	2755	0	24	6	6619	0.02
3365	3	89	24	2633	0	32	12	6037	0.05
3517	2	49	12	2600	1	14	7	6139	0.05
3487	1	96	43	2564	1	43	14	6110	0.03
3227	1	53	49	2457	1	33	34	5769	0.03

**Table 6:** Progeny of the strain T(Y:*bp*^+^)-7 during ten generations without removing recombinants

F	No. of pupae		Total pupae	Adults *bp*				Adults *bp^+^*				Total insects	Recombinants (%)
						
	*bp*	*bp^+^*		♀	♂	♀ partly emerged	♀ deformed	♂	♀	♂ partly emerged	♂ deformed		
P	935	1072	2007	709	0	20	3	1009	0	9	2	1718	0.00
F_1_	1058	1450	2508	1045	0	14	16	1311	0	15	5	2356	0.00
F_2_	1299	1348	2647	1142	0	16	9	1262	0	16	6	2451	0.00
F_3_	1185	1499	2684	1026	0	12	4	1410	1	12	8	2473	0.04
F_4_	1178	1198	2376	1051	0	6	3	1159	0	6	5	2230	0.00
F_5_	1135	1304	2439	856	0	12	8	1009	0	15	12	1912	0.00
F_6_	1294	1405	2699	919	0	146	49	1023	0	60	19	2216	0.00
F_7_	1134	1408	2542	849	0	33	7	1103	0	22	9	2023	0.00
F_8_	8000	7000	15000	5016	16	260	116	4313	14	102	48	9885	0.30
F_8_	834	968	1802	606	5	17	41	808	17	6	12	1512	1.50
F_9_	831	1188	2019	641	1	37	17	1027	9	7	4	1743	0.57
F_10_	938	1096	2034	713	0	12	3	986	13	6	3	1699	0.76

### Cytogenetic analysis

**Figure 2:**
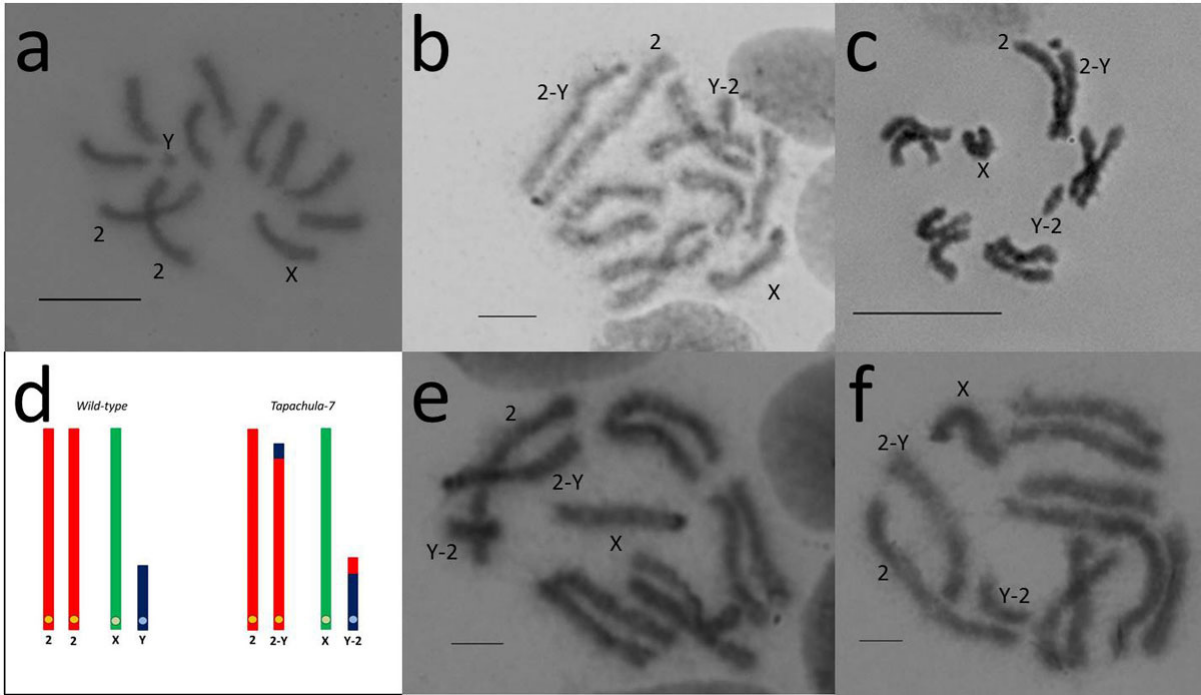
**a) *Anastrepha ludens *mitotic metaphase chromosomes from brain ganglia of third instar male larva of the wild-type strain**. b-c**) **Mitotic metaphases of the GSS *Tapachula-7*. d**) **Graphic representation of the Y-autosome translocation observed in the *Tapachula-7 *strain and its comparison with the wild-type. e-f) Mitotic metaphases of the second GSS, *Tapachula -1*. X, Y: represent the sex chromosomes, 2: represents the intact free autosome, Y-2: represents the translocation fragment with the Y centromere, 2-Y: represents the reciprocal translocation fragment carrying the autosomal centromere. Centromeres are indicated by circles in 2d. *Bar *= 10µm

**Mitotic chromosomes**. *Anastrepha ludens *has six pairs of acrocentric chromosomes, five autosomes and a pair of sex chromosomes (Figure 2a) [21].The male is heterogametic (XY) [21]. The autosomes display the characteristic somatic pairing observed in Diptera and this is very useful for the identification of homologous chromosomes. The analysis of at least 80 cells from the GSS T(Y:*bp*^+^)-7 indicates that the translocation involves the largest autosome (chromosome 2) and the Y chromosome. As it is evident from Figures 2b,c a small distal part of this autosome is joined to the Y chromosome, while a tiny part of the Y chromosome is connected to the autosomal breakpoint. The resulting 2-Y translocation chromosome is slightly shorter (10.58 ± 0.99%) than the wild-type chromosome 2 (11.93 ± 0.8%). The reciprocal Y-2 translocation fragment in the strain T(Y:*bp*^+^)-7 is longer than the wild-type Y chromosome (2.75 ± 0.37% versus 1.71 ± 0.3%). A graphic representation of the Y-autosome translocation is shown in Figure 2d.

The analysis of the mitotic chromosomes in another GSS strain, T(Y:*bp*^+^)-1, showed that the same autosome, chromosome 2, is involved in the translocation. However, the autosomal breakpoint seems to be at a different position because the 2-Y fragment was here 9.08 ± 0.62% and the Y-2 fragment 3.77 ± 0.31% long (Figure 2e-f).

**Figure 3:**
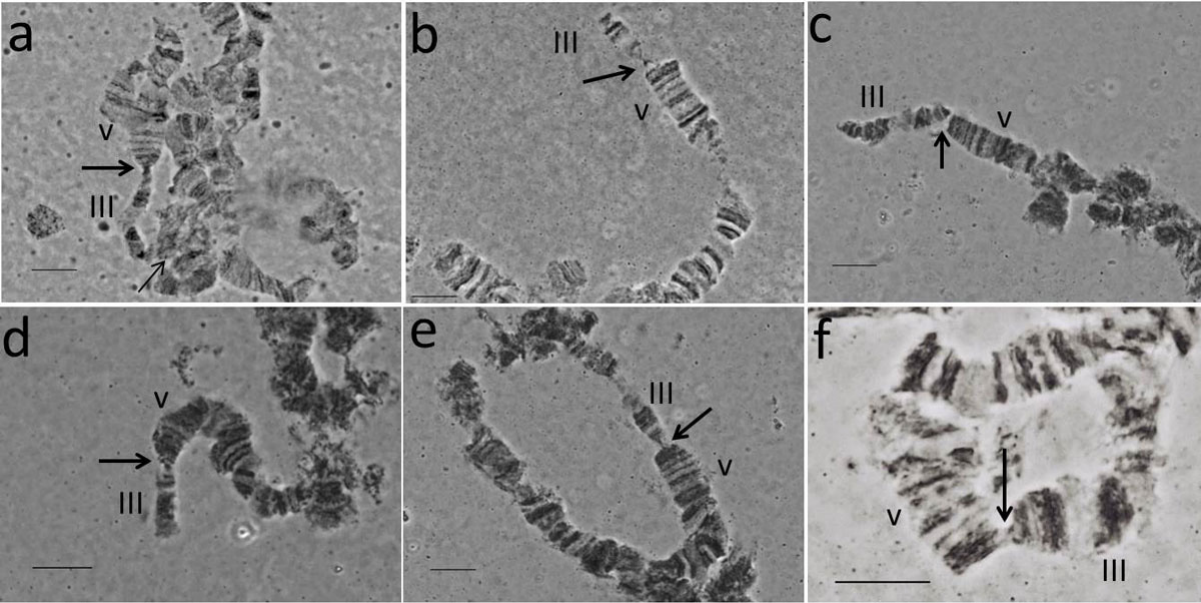
**Polytene chromosomes from the strain T(Y:*bp*^+^)-7**. Thick arrows show the ectopic paring between the telomeres of the V and III chromosomes. a) Thin arrow shows the autosome breakpoint; in this case the intact chromosome III is taken part in the ectopic pairing. b-e) The autosome fragment of the Y-A chromosome is taken part in the ectopic pairing. f) Normal ectopic pairing between chromosomes V and III from the wild-type strain. *Bar *= 10µm

**Figure 4:**
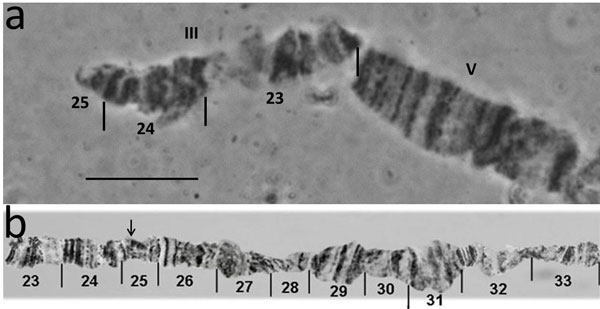
**a) The short translocation fragment representing the Y-2 chromosome of mitotic metaphases or Y-III of polytene chromosomes**. b) Reference map of chromosome III showing the region involved in translocation; arrow in the map shows the position of the breakpoint. *Bar *= 10µm

**Polytene chromosomes**. Polytene chromosomes were analyzed in male larvae from the GSS T(Y:*bp*^+^)-7. The analysis was focused only on males where the reciprocal translocation can be observed. Although the Y chromosome cannot be identified in polytene nuclei using standard staining, we succeeded to identify the autosome involved in the translocation and map the autosomal breakpoint. *Anastrepha ludens *shows a characteristic ectopic pairing between the telomeres of the autosomes [21], a phenomenon that also was observed in other Tephritidae species as *Bactrocera oleae *[22] and *B. cucurbitae *[23]. One of the most frequent ectopic pairing has been observed between the polytene elements III and V (Figure 3f). Polytene chromosomes from the strain T(Y:*bp*^+^)-7 are shown in Figure 3a-e. The ectopic pairing of the two telomeres, chromosome III and V are shown, but interestingly only one homolog of the polytene element III is involved in this. In all cases, a part of a single chromosome III is connected with its telomere to the telomere of chromosome V. This region should represent the distal part of the chromosome element involved in the Y-autosome translocation. In this ectopic pairing either the telomere of the intact chromosome III is taking part (Figure 3a); or the fragment of the chromosome that is connected to the Y chromosome (Y-A) (Figure 3b-e) is pairing. Based on these observations as well as on the mitotic karyotype of this strain, we can conclude that polytene chromosome III corresponds to the longest chromosome of the mitotic karyotype, i.e. chromosome 2. Taking into account both the mitotic karyotype and polytene chromosomes analyses of this GSS, we can propose the structure of the T(Y:*bp^+^*)-7. According to the chromosome maps of *A. ludens *[21] the chromosome III has a breakpoint at the beginning of the region 25 (Figure 4). This breakpoint produces two autosomal fragments of different size: The short fragment includes the distal part of the chromosome from the telomere to 25 chromosomal region (24.67 µm) while the second fragment consisting of the rest of the chromosome element III. The short fragment is joined to that part of the Y chromosome that carries the centromere Y-2 (Figure 2d). The second autosomal fragment, with the autosomal centromere, is linked to remaining region of the Y chromosome resulting in the 2-Ychromosome (Figure 2d) that is slightly shorter than the wild-type chromosome element III. Each of these new chromosomes, 2-Y and Y-2 has its centromere and telomere sufficient for their stability.

## Discussion

We have developed a GSS for *Anastrepha ludens *using the *black pupae *mutation as a selectable marker. In this strain, males emerge from wild-type (brown) pupae, whereas females emerge from mutant (black) pupae. This particular mutation has additional benefits because it is also expressed in the larval and the adult stage. This characteristic confers various benefits to this system, including greater reliability, avoiding the difficulties in the clear discrimination of the colours as it was reported by Rössler [24] for the genetic sexing system based on the marker *dark pupae *in *Ceratitis capitata*. Undoubtedly, the genetic marker *white pupae *found in other fruit flies provide better separation due to the greater contrast with the wild-type pupae [25-30].

However, despite significant efforts, this marker has not been found yet in *Anastrepha ludens*, neither in field populations nor in inbred laboratory colonies.

During this study, different doses of irradiation were used: 25 Gy, 30 Gy and 40 Gy. The first dose was too low, and no translocations were obtained. The highest dose induced such a high sterility that it was impossible to breed the insects. The optimal dose was 30 Gy, and the success rate was 0.38% (10/2600). The doses used to induce translocations were lower than those used in *Ceratitis capitata*. Franz et al., (1994) [31] reported a success rate of 7.1% at 50 Gy, and Kerremans and Franz, (1995) [17] reported a success rate of 4.3% at 40 Gy. These differences between *C. capitata *and *A. ludens *indicate that it is necessary to determine the optimal dose for each species individually. The optimal dose can be influenced by specific biological characteristics, such as the size of the Y chromosome. The Y chromosome of *C. capitata *is significantly longer than that of *A. ludens *and this might be responsible for a greater probability for inducing Y-autosome translocation compared to *A. ludens*.

Genetic stability is one of the most important aspects in the evaluation of a GSS and its usefulness for a practical application. Genetic stability depends directly on the structure of the translocation, i.e. it depends on the distance between the autosomal breakpoint of the translocation and the location of the genetic marker [31]. This distance is directly proportional to the frequency of recombination in this chromosomal region in the heterozygotic males of a sexing strain [13]. The recombinant flies show a reverse phenotype as compared to the normal, non-recombinant flies, i.e. recombination in the parental males produces black pupae males and brown pupae females. The percentage of recombinant flies during various generations was high in the strains T(Y:*bp*^+^)-2, T(Y:*bp*^+^)-3 and T(Y:*bp*^+^)-8. These strains were considered unstable. On the contrary, the strain T(Y:*bp*^+^)-7 showed no recombinants when reared at a smaller scale. However, the strain showed 0.025% of recombinants per generation when the scale of rearing was increased for several generations. Monitoring a larger number of insects over a longer period of time will provide additional results to predict the behaviour of this strain under mass-rearing conditions.

**Figure 5:**
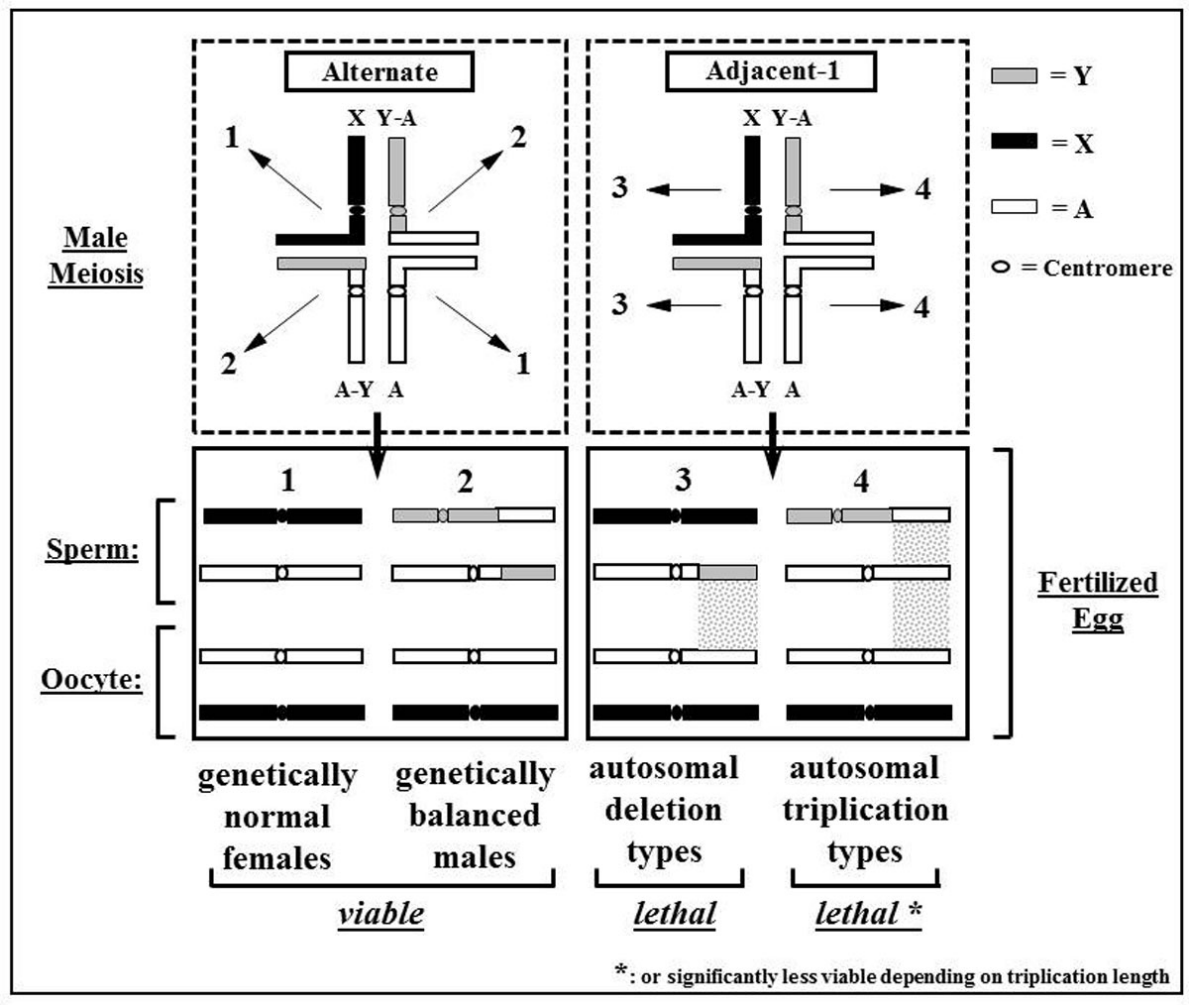
Schematic representation of alternate and adjacent-1 segregation in males with a Y-autosome translocation

In the evaluation of a GSS, it is important to consider the productivity and the product quality, both of which are tightly linked to the structure of the translocation. The segregation behaviour of the translocation during male meiosis will determine the number of genetically balanced or unbalanced individuals in the next generation. During male meiosis simple Y-autosome translocations can segregate in two different ways if they follow the rule that homologous centromeres segregate: alternate or adjacent-1 segregation. In the former the two translocation chromosomes stay together and segregate from the X chromosome. In the latter the A-Y chromosome segregates together with the X chromosome while the Y-A chromosome segregates with the non-translocated autosomal homologue. Figure 5 shows, that after fertilization only alternate segregation results in genetically balanced offspring while adjacent-1 segregation results in offspring where the chromosomal segment between the translocation breakpoint and the tip of respective chromosome arm is either present in three copies or segregation only in a haploid condition. At least in the medfly such long deletions lead to early lethality which is manifested as reduced egg hatch. The triplication type adjacent-1 offspring can survive, depending on the length of the triplication, even to the adult stage, although at severely reduced numbers [12]. Only if the autosome breakpoint is very close to the tip, i.e. the triplication is very short, such flies mate and produce offspring (Franz, personal communication). Both forms of segregation are completely legitimate and that explains why they occur at equal frequency in most translocation strains, at least in medfly. Consequently, only half of the sperm produced by males carrying a Y-autosome translocation lead to viable, genetically balanced offspring, i.e. such males are 50% sterile. In a third form of segregation, adjacent-2, homologous centromeres do not segregate, i.e. this is a form of non-disjunction and therefore significantly less likely to occur.

The egg hatch in strain T(Y:*bp*^+^)-7 is reduced due to the lethality of the deletion type adjacent-1 offspring. In addition, also the triplication carrying adjacent-1 individuals should ideally die as early as possible to avoid wasting larval diet and to avoid confusions in the quality control. In the T(Y:*bp*^+^)-7 strain, this seems to be the case as larval survival is the only parameter besides egg hatch that differs significantly from the wild-type level.

**Figure 6:**
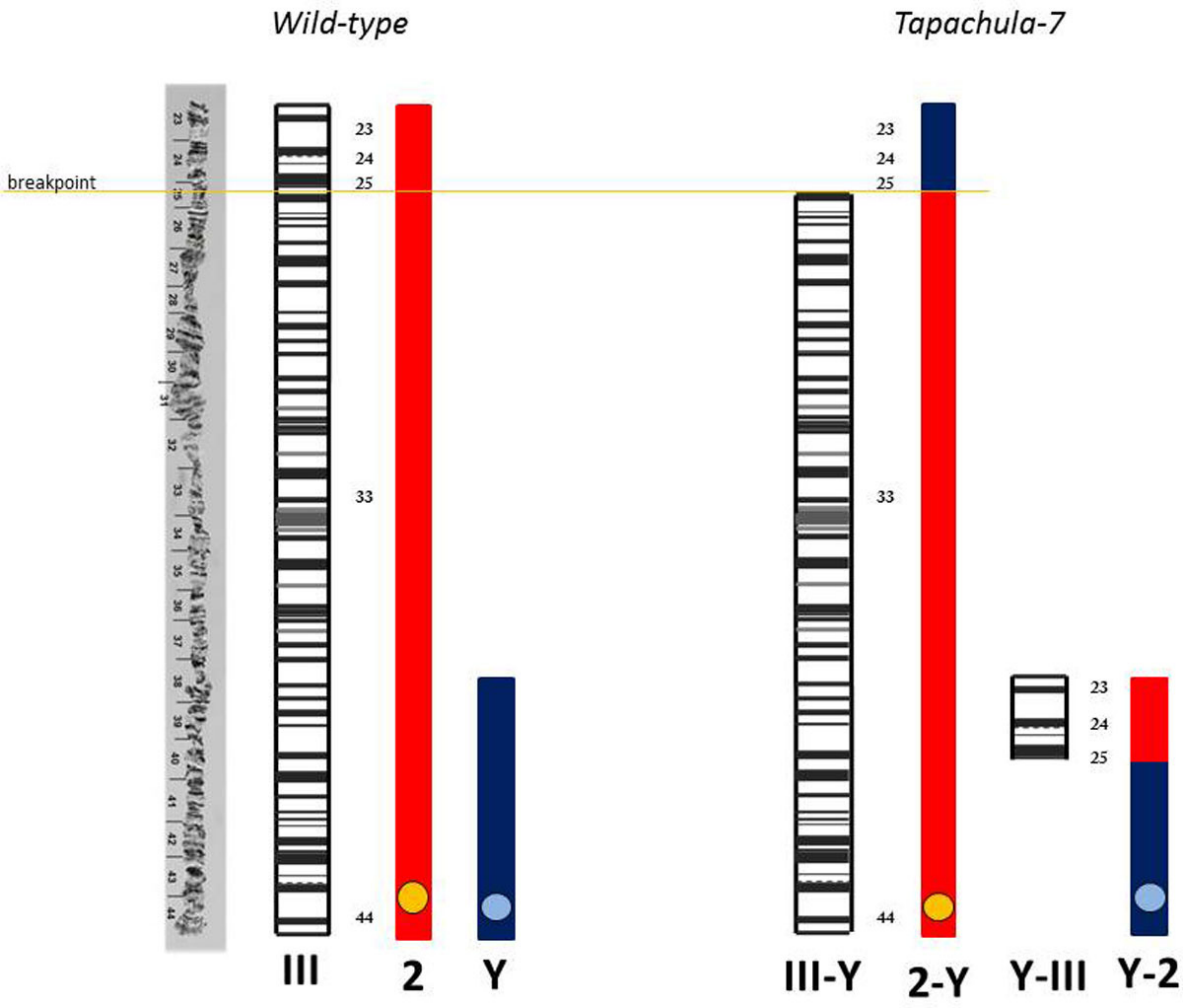
**Schematic representation of the correlation between mitotic chromosome 2 and polytene chromosome III in *wild-type *and *Tapachula-7 *strain showing the induced translocation in mitotic (2-Y) (Y-2) and the respective polytene chromosome (III-Y) (Y-III)**. Y represents sex chromosome.

The cytogenetic studies performed using mitotic chromosomes showed that T(Y:*bp*^+^)-7 carries a simple reciprocal translocation between the longest autosome, chromosome 2, and the Y chromosome. Analysis of polytene chromosomes permitted us to identify the polytene element involved in the translocation. Based on the specific ectopic pairing observed between the telomeres of autosomes in the *A. ludens *polytene nuclei [21], we show that chromosome 2 of the mitotic karyotype corresponds to polytene element III (Figure 6). In addition, this analysis permitted us to map the autosomal breakpoint on chromosome III despite the fact that the Y chromosome remains underreplicated in polytene nuclei and cannot be identified after standard staining. The autosomal breakpoint is close to the beginning of region 25 of chromosome III according to the polytene maps of the *A. ludens *(Figure 4). It should be emphasized that both translocation chromosomes possess centromeres and telomeres, required for stability during cell divisions.

Ideally, a genetic sexing system allows the elimination of females during the early stages of development to decrease the cost during mass-rearing effectively and to increase the capacity of the production facility. However, the method of sex separation we report here is based on the coloration of the pupae and allows sexing only at the pupal stage. This is done mechanically using sorting machines available on the market. These machines can be adapted for this specific use by modifying their sorting characteristics so that brown and black pupae can be separated with the required speed and accuracy. In 1969, Whitten [32] suggested this type of mechanized separation for GSS based on the colour of pupae to obtain sufficient quantities to meet the demand of an area-wide control program. The advantage of this method of separation is that it is non-destructive, allowing to feed the females back into the rearing colony and, thereby, increasing production capacity if required [33]. Using such a seed sorting machine, Robinson and Riva [34] separated white and brown pupae of a Mediterranean fruit fly GSS with a success rate of 100%.

## Conclusions

We have isolated the first GSS in *A. ludens*, which was given the common name *Tapachula-7*. Its characteristics, as far as we have determined them in the research described here, indicate that this strain will be stable and productive enough for a practical application in the mass-rearing for field operations. However, further testing under mass-rearing conditions is required to come to a final conclusion. In addition, the results of the current study show that mitotic and polytene chromosomes of this species are suitable for cytogenetic studies and could support the development of improved control methods in this pest.

## Materials and methods

### Biological material

The colony of *Anastrepha ludens *used in this study was founded using flies collected in the wild in the state of Chiapas, México [35]. The insects were then kept in the laboratory for several generations with photoperiods of 12:12, a temperature of 26°C and a relative humidity of 70-80%.

### *black pupae *strain

Pupae with black colour, distinct from the brown-coloured wild-type pupae, were isolated from the mass-rearing facility Moscafrut located in Metapa de Domínguez Chiapas, México after screening approximately 7, 700 000.00 pupae. Male and female adults derived from *black pupae *had noticeably darker cuticles and wing lines than the wild-type individuals. Larval anal lobes were black and easy to be differentiated from the wild-type [36].

### Rearing the flies

Adults are maintained in laboratory conditions at 26°C and a relative humidity of 60-70% with *ad libitum *water and a mixture of sugar and hydrolyzed proteins (yeast hydrolysate enzymatic MP Biomedicals, LLC France). Eggs are collected and incubated in Petri dishes with saturated humidity for 4 days at 26°C in a bioclimatic chamber Binder KBF720. The larvae are fed with diet of powdered corncob (19% powdered corncob, 9.2% sugar, 5.3% corn flour, 7.0% yeast, 0.44% citric acid, 0.4% sodium benzoate, 0.2% nipagin and 0.1% guar gum) and are maintained under controlled temperature and humidity. Pupation is carried out over 14 days on Vermiculite Strong Lite^® ^substrate.

### Genetic analysis

The inheritance of the *black pupae *mutation was determined by crossing wild-type males to *black pupae *females and *black pupae *males to wild-type females (♂wt to ♀*bp*, ♂*bp *to ♀wt). Three cages were set up for each cross. Eggs were collected from each cage, and the breeding process was continued until the emergence of the F_1 _generation progeny. Five females and 5 males of the F_1 _generation were then inbred to obtain the F_2 _generation. F_1 _and F_2 _pupae were carefully checked and separated by colour, and the adults were observed under a Zeiss stereomicroscope to confirm their phenotype.

### Development of genetic sexing strain

Wild-type pupae two days before emergence were irradiated with 30 Gray (Gy) using a cobalt-60 Gammacell 220 source. The irradiated males were then placed in 4 litre cages and crossed with virgin females homozygous for *black pupae *(*bp/bp*).

**Figure 7:**
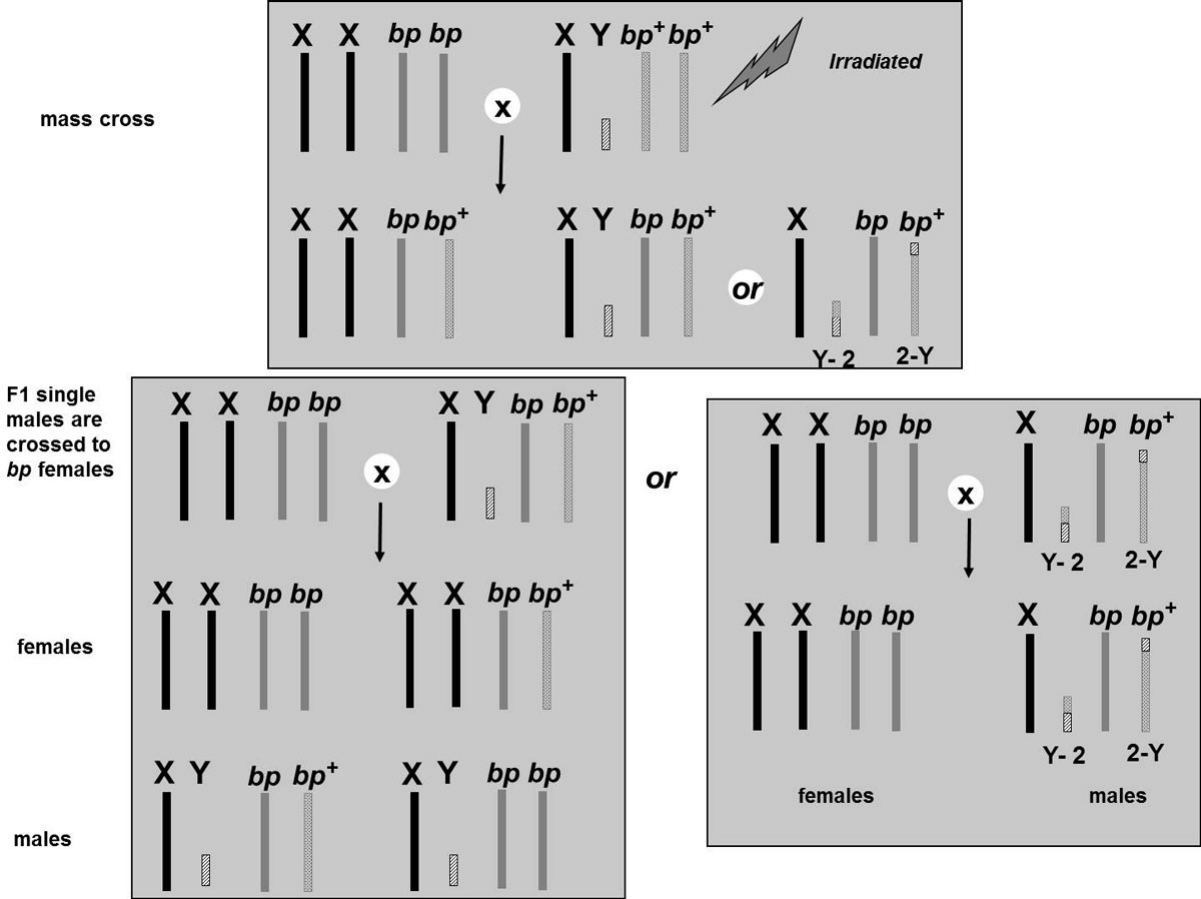
**Crossing scheme for the isolation of genetic sexing strains in *A. ludens*.** X and Y: represent the sex chromosomes. *bp^+^*: represents the autosome harboring the wild type allele for the brown pupae color. *bp*: represents the autosome harboring the *black pupae *(*bp*) mutation. Y-2 and 2-Y: represent the two new chromosomes following the translocation event.

The F_1 _males of that cross were singly backcrossed to mutant females. If the F_1 _males carry a translocation between the Y and the autosome bearing the selectable marker "*bp*", all the males of a particular family will be wild-type, and all the females will be mutant (Figure 7). Where such a family was detected in the screen, the progeny was further propagated, and their true-breeding nature was ascertained and maintained in the laboratory for several generations. In each generation, the percentage of the hatched eggs, the number of pupae and their colour as well as the number of adults were recorded. When recombinants, i.e. females emerging from brown and males from black pupae, were detected they were counted and removed. The pupal colour separation was performed manually, and the correct classification was corroborated in adults using the dark coloration of the *black pupae *strain for differentiation.

### Survival test and genetic stability

The following three types of crosses were set up: a) flies from the *black pupae *strain (100♀ and 100♂) b) wild-type (50♀ and 50♂) and c) the GSS *Tapachula-7 *(100♀ and 100♂). Each one was placed separately in cages of 30x30x30 cm. When the insects reached sexual maturity, 1000 eggs were taken in batches of 100. The eggs were incubated for 4 days at 26 ± 1°C in a bioclimatic KBF720 Binder chamber, and the number of fertile eggs was quantified after 7 days. The larval development of 100 eggs was conducted in a Petri dish with the specified larval diet. The number of third-instar larvae was quantified after 10 days. The number of mature pupae and emerged adults was also recorded.

The T(Y:*bp*^+^)-7 was reared for some generations increasing the number of parents. Three experiments were carried out: the first was using a population in the cage of 600-2500 adults, evaluated during seven generations and in each generation the recombinants were removed and the pupae yielded were recorded. In the second experiment the population used was 3000 females × 2500 males and recombinants were monitored during eight generations, removing the recombinants in each generation. In the third experiment we monitored ten generations of rearing with 15,000 parental pupae (mixed black and brown) per generation in large cages (the recombinants were not removed during this experiment). To determine the number of recombinants in each generation, a sample of 100 ml of pupae was analyzed in each generation.

### Cytogenetic analysis

Mitotic and polytene chromosomes were prepared from the genetic sexing strain T(Y:*bp*^+^)-7. Metaphase chromosomes were prepared from the brain ganglia of third-instar larvae [20] while for the staining the application of the C- banding technique was used [37].

The slides were immersed in hydrochloric acid 0.2 N for 90 s, washed with distilled water and immersed in 5% barium hydroxide at 40°C for 1 min. The slides were then washed in distilled water with a few drops of acetic acid and placed in 2xSSC at 60°C for 30 min. Finally, the slides were washed and stained using 25% Wright stain in Sörensen buffer for 1 min and 12% Giemsa stain for 1 hour. The samples were observed using a Zeiss Axioskop 40 microscope with 100x magnification objective, and images were acquired using the software Axiovision 4.8.2 by Carl Zeiss de México S.A. de C.V. The length of the chromosomes in 35 metaphases from the wild-type samples and 15 from the genetic sexing strain were measured with UTHSCSA Image tool 3.0 (web site http://compdent.uthscsa.edu/dig/itdesc.html). Only well metaphase spreads with 12 chromosomes were used to measure the total length of each chromosome. The relative chromosomal length was estimated as the percentage of the length of each chromosome relative to the total length of the total diploid complement (%RL). Third instar male larvae were used for the salivary gland polytene chromosomes using the method described for *C. capitata *[18,38] and previously used for *A. ludens *by García-Martínez et al. [21]. The male larvae were identified based on the brown coloration of the anal lobes compared to the black coloration of the female larvae.

### Data analysis

The overall fitness was estimated by multiplying the transformation frequency between the stages (proportion of eggs hatched, larvae survival, pupae survival and pupae to adult survival). The data were transformed by arcsine √x and analyzed by ANOVA. Medians were compared using Tukey's test with α = 0.5. Both of these analyses were performed using the software JMP version 5.0 (web site http://www.jmp.com).

## Competing interests

The authors declare that they have no competing interests.

## Authors' contributions

CSZC directed the study, designed the protocol, selected the data to present, integrated and composed the manuscript and collaborated with VGM to implement novel technical modifications for the study of mitotic chromosomes.

JSMH designed protocols for the construction and testing of the genetic sexing strains, integrated and analyzed the data, performed the statistical analysis and help to integrate the initial draft of the manuscript.

VGM conducted the laboratory cytogenetic methods, including slide preparation and imaging, discussed the results of the cytogenetic analysis and revised the manuscript.

JIP coordinated the laboratory work for establishing protocols and evaluating the genetic sexing strains.

AZ participated in the interpretation of the cytogenetic analysis data and reviewing the manuscript.

GF designed the protocols for constructing the genetic sexing strains and reviewing the manuscript.
